# Trypanocidal Activity of Marine Natural Products

**DOI:** 10.3390/md11104058

**Published:** 2013-10-22

**Authors:** Amy J. Jones, Tanja Grkovic, Melissa L. Sykes, Vicky M. Avery

**Affiliations:** Eskitis Institute for Drug Discovery, Griffith University, Nathan, Brisbane 4111, Australia; E-Mails: a.jones@griffith.edu.au (A.J.J.); t.grkovic@griffith.edu.au (T.G.); m.sykes@griffith.edu.au (M.L.S.)

**Keywords:** human African trypanosomiasis, sleeping sickness, chagas disease, marine natural products, drug discovery

## Abstract

Marine natural products are a diverse, unique collection of compounds with immense therapeutic potential. This has resulted in these molecules being evaluated for a number of different disease indications including the neglected protozoan diseases, human African trypanosomiasis and Chagas disease, for which very few drugs are currently available. This article will review the marine natural products for which activity against the kinetoplastid parasites; *Trypanosoma brucei brucei*, *T.b. rhodesiense* and *T. cruzi* has been reported. As it is important to know the selectivity of a compound when evaluating its trypanocidal activity, this article will only cover molecules which have simultaneously been tested for cytotoxicity against a mammalian cell line. Compounds have been grouped according to their chemical structure and representative examples from each class were selected for detailed discussion.

## 1. Introduction

The trypanosomatid diseases human African trypanosomiasis (HAT) and Chagas disease account for over 19,000 deaths and the loss of over 100,000 disability adjusted life years (DALYs) annually [1,2]. The etiological agents of the disease are kinetoplastid parasites of the genus *Trypanosoma*. *Trypanosoma brucei gambiense* and *Trypanosoma brucei rhodesiense* are responsible for HAT, while infection with *Trypanosoma cruzi* is the causative agent of Chagas disease. Both diseases rely on insect vectors for their transmission; tsetse flies (*Glossina* spp.) are the vectors for HAT, whereas a number of *Triatoma* bug species transmit *T. cruzi* [3,4]. HAT is prevalent throughout 36 sub-Saharan African countries whilst Chagas disease primarily occurs in Southern parts of North America, and South America [5,6].

Initially, inoculation of the parasites into human hosts results in acute disease. In HAT, this is characterized by the presence of the parasites in the vasculature and lymphatic systems. Patients experience fever, nausea, headaches and lymphedema [7]. Without treatment the parasites penetrate the blood brain barrier (BBB) and invade the central nervous system (CNS) initiating chronic or CNS stage disease. CNS stage disease manifests as mental disturbances, anxiety, hallucinations and a characteristic disruption of the sleep-wake cycle [7,8,9,10]. Without treatment the disease is considered fatal [11].

In contrast to HAT, acute Chagas disease is often asymptomatic and as such is not often diagnosed [12]. Approximately one third of infected individuals go on to develop the chronic form of the disease which can remain asymptomatic for 10 to 30 years [12]. The chronic stage can manifest as cardiac or cardiodigestive disorders (megacolon, megaeosphagus), or a combination of these [13]. Chagas related heart disease is one of the major causes of morbidity and mortality in endemic areas [14].

**Figure 1:**
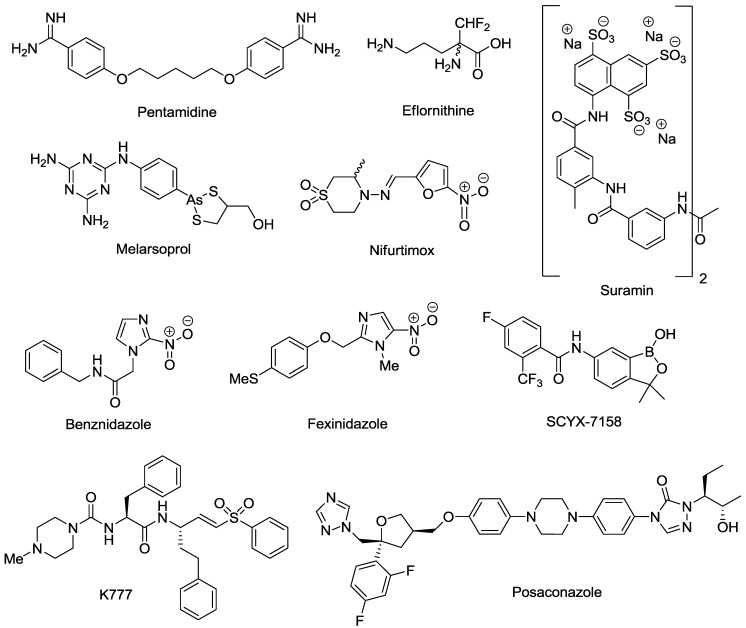
Drugs currently registered and in development for the treatment of human African trypanosomiasis (HAT) and Chagas disease.

Despite the morbidity and mortality inflicted by HAT and Chagas disease, very few effective drugs are currently available (Figure 1). Acute *T.b. gambiense* and *T.b. rhodesiense* infections are treated with pentamidine and suramin, respectively [15]. CNS *T.b. rhodesiense* infections are treated with melarsoprol, while *T.b. gambiense* infections are treated with either eflornithine or a nifurtimox/eflornithine combination therapy (NECT) [15]. However, none of these treatments are ideal. Melarsoprol is extremely toxic, resulting in the death of 5% of all patients to whom the drug is administered, and eflornithine has a complicated, protracted administration schedule requiring 56 slow intravenous (i.v.) infusions over 14 days [16,17]. The development of NECT reduced the administration schedule of eflornithine to 14 i.v. infusions over seven days, plus oral nifurtimox every eight hours for 10 days [18,19]. However, NECT is not ideal as parenteral administration is still required and patients must be hospitalized for the duration of treatment. Acute and chronic Chagas diseases are treated with either nifurtimox or benznidazole. Both drugs have lengthy administration schedules requiring bi- or tri-daily administration for 60 to 90 days [20]. Patients frequently experience vomiting, nausea, hepatic intolerance, convulsions and skin disease manifestations [21]. The unpleasant side effects experienced by patients, coupled with administration schedules, result in many patients failing to complete the treatment regimes [22,23].

The paucity of safe, effective and easily administrable drugs for HAT and Chagas disease is partly due to a lack of interest by large pharmaceutical companies. HAT and Chagas disease primarily affect poor, disadvantaged people, with limited access to health care and very little means to pay for drugs. Consequently, there is little incentive for pharmaceutical companies to invest in the research and development of new compounds for these disease indications. It has only been in the last decade, with the establishment of non-for-profit organizations such as the Drugs for Neglected Diseases initiative (DND*i*) and the Bill and Melinda Gates Foundation, that substantial investment and progress has been made in drug discovery for HAT and Chagas disease. As a result, one compound, fexinidazole, is now in phase II/III clinical trials for HAT, while a second compound, SCYX-7158, is in phase I clinical trials [24,25]. In addition, during the past five years numerous drug targets have been identified and validated in *T.b. brucei* which are discussed in detail in a recent review [26]. Promising targets described include, the enzymes S-adenosylmethionine decarboxylase (AdoMetDC) [27,28], N-myristoyltransferase (NMT) [29,30] and trypanothione synthetase-amidase (TrySyn) [31]. For Chagas disease, K777 is currently in pre-clinical trials [32], whilst clinical trials with posaconazole are due for completion in 2013 [33]. Target identification studies have indicated that cysteine protease is the target of K777, thus validating further development of this class of inhibitors. Posaconazole inhibits *T. cruzi* sterol 14α-demethylase (CYP51) [34], and research continues to identify further inhibitors of this specific target [35,36,37]. Azole antifungals with CYP51 activity have previously entered clinical trials, however, have not demonstrated curative activity [38]. Few validated targets have been identified against *T. cruzi* and studies to determine new targets will be of benefit for Chagas disease research. Cloning of recombinant proteins based on the identified genome sequence could facilitate this process [39]. The mitochondria and mitochondrial metabolism [40] have been identified as potential sources of new targets for *T. cruzi* drug discovery research, as well as enzymes involved in pentose phosphate and thymidine synthesis [41].

Non-for profit organizations have highlighted the plight of HAT and Chagas disease patients and have provided the financial resources required for new therapeutics to be identified and developed. However, numerous problems still exist which impede drug development for HAT and Chagas disease. A large proportion of the molecules identified by phenotypic high-throughput screening (HTS) campaigns have undesirable chemical properties and biological characteristics, which makes them unsuitable for further development. Structure activity relationship (SAR) studies are frequently undertaken in order to improve a molecule’s physiochemical properties, but this often results in a significant loss of trypanocidal activity. In the last five years, multiple drug targets have been identified in *T. brucei* spp. and *T. cruzi*. However, the targets are often inaccessible and it is difficult to develop small molecule inhibitors, which are capable of reaching and interacting with the target. Target-based screening can be utilized to identify potent inhibitors of targets but often the molecules lack trypanocidal activity when subsequently screened against the whole parasite, as they are unable to penetrate the parasites and reach the intracellular target.

The high attrition rate associated with drug discovery and development and the difficulties encountered, means that there still exists a critical need to identify novel compounds for HAT and Chagas disease. Natural products including, marine organisms and metabolites, are one potential source from which unique trypanocidal compounds could be identified.

Natural products are attractive chemical starting points for drug discovery. They have been investigated for a number of different disease indications and biological targets resulting in the identification of both lead molecules and drugs suitable for entry into the drug discovery pipeline. Between 1981 and 2010 natural products and synthetic small molecules either derived from a natural product or based on a natural product, pharmacophore, accounted for over 50% of new chemical entities [42]. Research into the chemistry, pharmacology and therapeutic potential of marine natural products began with the development of self-contained breathing apparatus (SCUBA) in the 1960s and has continued to progress and develop with thousands of compounds now identified [43]. The first marine natural product to be registered by the United States (US) Food and Drugs Administration (FDA) was cytarabine (1β-arabinofuranosylcytosine), a chemotherapeutic agent, in 1969. Since then six other marine natural product based drugs have been approved by the FDA; vidarabine (anti-cancer and anti-viral), ziconotide (an analgesic agent), eribulin mesylate (anti-cancer), brentuximab vedotin (for the treatment of Hodgkin’s lymphoma and large cell lymphoma) and the omega-3-ethyl ester preparations, lovaza and vascepa (triglyceride lowering agents). In addition, one further compound, trabectedin (anti-cancer), has been approved by the European Medicines Agency (EMA).

**Figure 2:**
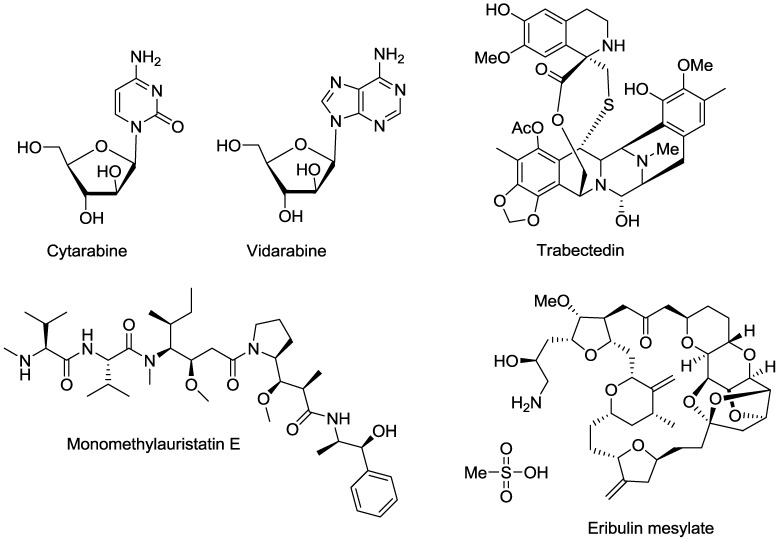
Examples of small molecule-based marine natural products or their derivatives which have received Food and Drug Administration (FDA) or European Medicines Agency (EMA) approval.

Cytarabine (1β-arabinofuranosylcytosine) and vidarabine (adenine arabinoside) (Figure 2) are synthetic pyrimidine and purine nucleosides, respectively, developed from nucleosides isolated from the Caribbean sponge *Tethya crypta* [44,45]. Cytarabine is used for the treatment of acute myeloid and lymphocytic leukemia, while vidarabine was approved in 1976 for the treatment of acute keratoconjunctivitis and recurrent epithelial keratitis caused by *Herpes simplex* viruses [46,47,48]. The therapeutic effects of cytarabine and vidarabine are thought to arise due to inhibition of DNA polymerase and DNA synthesis [49,50].

Twenty-eight years after the registration of vidarabine, ziconotide, a synthetic equivalent of a peptide originally isolated from the venom of the cone snail *Conus magus*, was approved by the FDA [51]. The drug is a powerful analgesic due to its ability to selectively and specifically block N-type voltage sensitive calcium channels and is used to manage chronic pain in cancer and AIDS patients [52]. Also in 2004, lovaza, the first drug containing the fish derived omega-3-ethyl fatty acids, eicosapentaenoic acid (EPA) and docosahexaenoic acid (DHA) was approved for the reduction of triglyceride levels in severe hypertriglyceridemia [53]. This was followed by the registration of vascepa, containing only EPA, in 2012 [54]. Omega-3-ethyl fatty acids are found in all fish species but are most abundant in oily fish, such as salmon, mackerel and herring [55]. The mechanism of action (MOA) for the hypotriglyceridemic effect of omega-3-ethyl fatty acids is not fully understood but has been attributed to the suppression of hepatic lipogenesis, an increase in fatty β-oxidation and down regulation of hepatic nuclear factor-4α (HNF-4α) [56,57,58,59,60]. In 2010, eribulin mesylate, a synthetic macro-cyclic ketone analogue of halichondrin B, a molecule isolated from the marine sponge *Halichondria okadai*, received FDA approval for the treatment of metastatic breast cancer [61]. Eribulin induces cell death by inhibiting microtubule growth and sequestering tubulin into nonproductive aggregates [62,63,64]. Brentuximab vedotin, a CD30 specific antibody-drug conjugate received FDA approval for the treatment of Hodgkin’s lymphoma in 2011. Brentuximab vedotin is composed of monomethylauristatin E (MMAE), a synthetic analogue of the marine natural product dolastatin 10 conjugated with the chimeric anti-CD30 monoclonal antibody, SGN-30 [65]. Dolastatin 10 was originally isolated from the Indian Ocean sea hare *Dolabella auricularia* in 1987 [66]. MMAE is an anti-tubulin agent which binds to tubulin and prevents microtubule polymerization leading to G_2_-M phase growth arrest and apoptosis [67]. Trabectedin (ecteinascidin) (Figure 2) has been approved in the European Union (EU) by the EMA. The compound was isolated from the ascidian *Ecteinascidia turbinata* and is an anti-cancer agent used in the treatment of soft tissue sarcoma and platinum-sensitive ovarian cancer [68]. The MOA of trabectedin is not fully elucidated, however, the compound has been shown to bind to the minor groove of DNA and interact with different binding proteins of the Nucleotide Excision Repair System (NERS) [69,70,71,72]. In addition to the marine natural products which have received regulatory approval and progressed to the market, numerous molecules are currently in clinical development [73].

To date, no marine natural products or derivatives have entered pre-clinical development specifically for trypanosomatid diseases. However, numerous marine natural products which exhibit anti-trypanosomal activity have been reported in the literature.

**Figure 3:**
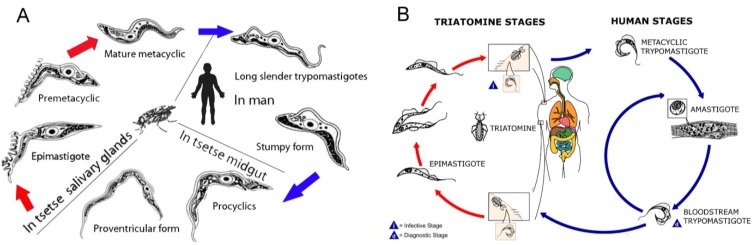
(**A**) The life cycle of *T. brucei* spp. (modified content from [82]). (**B**) The life cycle of *T. cruzi* (modified content from [83]).

In this article, the natural products isolated from marine sources for which activity against the protozoan parasites; *T.b. brucei*, *T.b. rhodesiense* and *T. cruzi* has been reported, is reviewed. The majority of the compounds have been identified through phenotypic screening campaigns, which have recently been reviewed in detail [74,75]. It should be noted that although *T.b. brucei* primarily infects domestic mammals and antelopes and is not the human infective subspecies responsible for HAT, it is frequently used in early drug discovery screening campaigns to identify active compounds [76,77]. Compounds active against *T.b. brucei* would ultimately be evaluated against the human infective forms of the parasite, *T.b. rhodesiense* and *T.b. gambiense*. The bloodstream form of *T. brucei* spp. is used in phenotypic screening assays, as this is the clinically relevant form of the parasite (Figure 3A). In *T. cruzi* infection, the amastigote and the trypomastigote life cycle stages are both found within the human host (Figure 3B). All lifecycle stages of *T. cruzi* can be used in assays to evaluate the activity of compounds. However, activity against the amastigote form of the parasite has been deemed to be of primary importance in many assays, with activity against the trypomastigote stage also considered favorable or necessary [78,79,80]. Herein, only activity against the human infective forms, namely amastigotes and trypomastigotes are considered. Many assay formats used in *T. cruzi* research are based on the method by Buckner *et al.*, whereby compounds are added two hours after addition of *T. cruzi* β-galactosidase transfected trypomastigotes to host cells [81]. Cells are incubated for seven days before detection of released trypomastigotes via lysis of cells and detection of β-galactosidase activity. This assay may affect both host cell infection and/ or development of amastigotes. The *T. cruzi* assays discussed in this article are based on this assay format, unless a modification is discussed.

In drug discovery screening campaigns for HAT and Chagas disease, a compound is only classed as a “hit”, if it has an IC_50_ < 10 µM [84,85,86,87]. Compounds with an IC_50_ ≥ 10 µM would not be considered suitable for progression along the drug discovery pipeline and would only be used as tools or probes. In this review, the trypanocidal activity of compounds is described according to their IC_50_ values and are defined as: IC_50_ < 10 µM = promising trypanocidal activity, 10 µM ≤ IC_50_ < 20 µM = moderate activity, 20 µM ≤ IC_50_ < 30 µM = marginal activity, 30 µM ≤ IC_50_ < 40 µM = limited activity, IC_50_ ≥ 40 µM = no activity/inactive. When evaluating the activity of compounds against a human pathogen or disease target it is important that the cytotoxicity of the compound is also investigated against a mammalian cell line to allow the selectivity index (SI) of the compound to be determined. The SI for selecting compounds with anti-trypanosomal activity is the ratio of the IC_50_ value obtained for mammalian cells divided by the IC_50_ against trypanosome species. We have considered herein that an SI < 10 suggests that the compound may be exerting a generally toxic effect. If the SI is ≥10, the compound is considered to have some selective activity against the parasite. However, a significantly greater SI is required in order for molecules to progress along the drug discovery pipeline and eventually into clinical studies.

This article will focus on compounds which have an IC_50_ < 40 µM against *T.b. brucei*, *T.b.* *rhodesiense* or *T. cruzi* and which have also been evaluated against a mammalian cell line. Compounds have been grouped according to their chemical structures into three categories; terpenes, polyketides and xanthones, and alkaloids. Representative examples for each category are discussed in terms of their trypanocidal activity and SI. To allow the activity of compounds to be compared independently of their molecular weight, all literature values have been converted into micromolar concentrations (µM).

## 2. Marine Natural Products with Reported *in Vitro* Activity against the *Trypanosome* Species *T. cruzi*, *T. brucei* or *T.b. rhodesiense*

### 2.1. Terpenes

**Figure 4:**
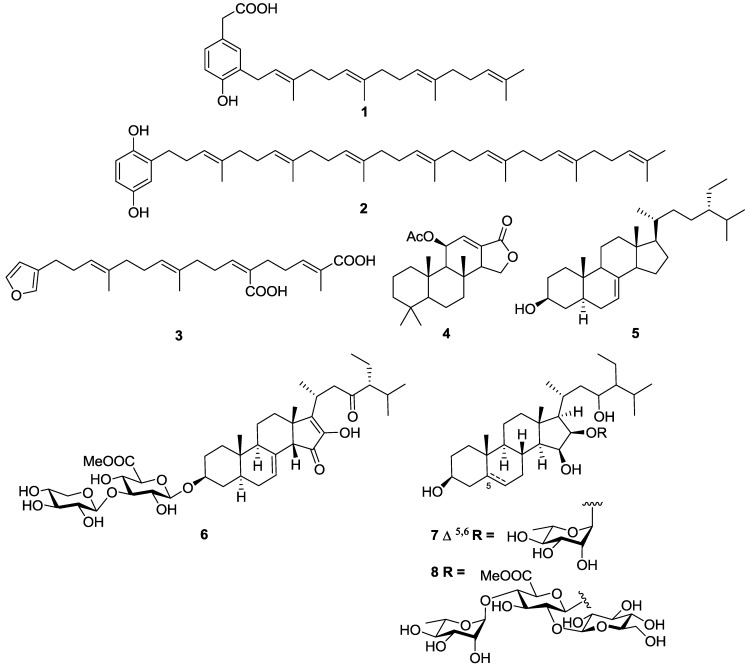
The structure of terpenes of marine origin which have exhibited activity against *T.b. rhodesiense* (**1**–**8**) and *T. cruzi* (**6**–**8**).

The marine sponges *Spongia* sp. and *Ircinia* sp. collected from the Turkish coastline of the Aegean Sea yielded a series of linear furanoterpenes and meroterpenes, as well as di- and tri-terpenes all of which were assessed for growth inhibitory activity against a series of protozoan parasites (Figure 4) [88]. 4-hydroxy-3-tetraprenylphenylacetic acid (**1**) was the most active and selective molecule with an IC_50_ value of 1.4 µM against *T.b. rhodesiense* and a selectivity index (SI) of >150, *versus* mammalian L6 rat skeletal muscle cells. The related structure heptaprenyl-*p*-quinol (**2**) possessing a longer isoprene chain and a hydroquinone terminal unit showed promising activity against *T.b. rhodesiense* with an IC_50_ value of 5.9 µM, however had no selectivity with an almost equivalent IC_50_ value of 4.4 µM observed against L6 cells. Demethylfurospongin-4 (**3**) was selectively active against *T.b. rhodesiense* with an IC_50_ value of 11.8 µM and an SI > 18. The diterpene 11β-acetoxyspongi-12-en-16-one (**4**) exhibited moderate activity against *T.b. rhodesiense* with an IC_50_ value of 11.5 µM but had no selectivity with an IC_50_ of 9.2 µM against L6 cells [88]. A number of trypanocidal molecules with varying degrees of activity have been identified from *Agelas* sp. marine sponges. The sterol 24-ethyl-cholest-5α-7-en-3-β-ol (**5**) isolated from the *n*-hexane extract of the Turkish sponge *Agelas oroides* showed limited activity against *T.b. rhodesiense* with an IC_50_ value of 34.2 µM [89]. Compound **5** was inactive against both *T. cruzi* (IC_50_ > 72 µM) and L6 cells (IC_50_ > 217 µM). These authors used the *T. cruzi* β-galactosidase assay to estimate compound activity [81].

A series of steroidal saponins characterized by a 2-hydroxycyclopentenone ring D and a glucuronic acid substituent at C-3 isolated from the Caribbean sponge *Pandaros acanthifolium* have demonstrated wide-ranging biological activity, including inhibition of both *T.b. brucei* and *T. cruzi*. Notably, pandaroside G methyl ester (**6**) had sub-micromolar activity against both *T.b. rhodesiense* and *T. cruzi* with IC_50_ values of 0.038 and 0.77 µM, respectively [90]. However, the molecule was not specific for *T.b. rhodesiense* or *T. cruzi* as it also inhibited mammalian L6 cells with an IC_50_ value of 0.22 µM, suggesting the natural product was generally toxic. Related steroidal saponins, the acanthifolisides, were also isolated as minor components from the same sponge collection [91]. Acanthifolioside E (**7**) showed moderate activity against *T. cruzi*, with an IC_50_ value of 10.6 µM, and marginal activity against *T.b. rhodesiense*, with an IC_50_ of 27.4 µM. In contrast, the trisaccharide acanthifolioside F methyl ester (**8**) had promising activity against *T.b. rhodesiense* with an IC_50_ value of 6.4 µM but only displayed marginal activity against *T. cruzi*, IC_50_ = 22.2 µM. Both compounds showed pan-panel activity against a series of other protozoa, as well as low SI values (<3) against mammalian L6 cells.

### 2.2. Polyketides and Xanthones

**Figure 5:**
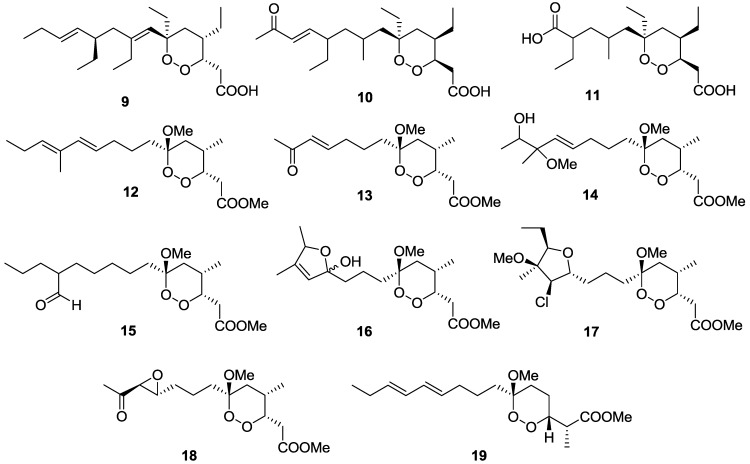
The structure of endoperoxide polyketides of marine origin for which activity against *T. cruzi* (**9**), *T.b. brucei* (**10**–**11**), and *T.b. rhodesiense* (**12**–**19**) has been described.

A series of marine-derived polyketide endoperoxides have shown potent activity and good selectivity against trypanosomes (Figure 5). Plakortide P (**9**) isolated from a Brazilian collection of the sponge *Plakortis angulospiculatus* inhibited *T. cruzi* with an IC_50_ value of 6.3 μM but had a poor SI of 7 [92]. These authors utilized the soluble tetrazolium salt, MTT, to detect the metabolic activity of host cell-free trypomastigotes. 11,12-didehydro-13-oxo-plakortide Q (**10**) and 10-carboxy-11,12,13,14-tetranor-plakortide Q (**11**) isolated from an Australian collection of the sponge *Plakortis* sp. showed activity against *T.b. brucei* with IC_50_ values of 0.049 and 0.940 μM, respectively, and favorable selectivity indices, with compound **10** displaying a SI of 105 times and compound **11** <88 times over the human embryonic kidney cells, HEK-293 [93]. Interestingly, a substitution of the enone functionality in **10** with that of a carboxylic acid group in **11** resulted in a 20-fold reduction of activity against *T.b. brucei*. Related structures, manadoperoxides and peroxyplakoric ester B3 isolated from the Indonesian sponge *Plakortis* cfr. *lita* were also found to inhibit *T.b. rhodesiense* at low micro-molar concentrations [94]. Manadoperoxides B (**12**), C (**13**), F (**14**), H (**15**), I (**16**), and K (**17**) exhibited IC_50_ values of 0.0088, 2.2, 2, 1, 0.17, and 0.2 μM respectively, with favorable selectivity indices of > 3000, >15, >13, >27, >161 and >115, against human mammary epithelial cells (HMEC). Manadoperoxide G (**18**) as well as the peroxyplakoric ester B3 (**19**) were demonstrated to have moderate activity against *T.b. rhodesiense* with IC_50_ values of 5.6 and 11 µM, but exhibited very poor selectivity (< 5). The availability of ten structurally related analogues of manadoperoxide B gave an insight into the structure-activity relationship for this chemical class of compounds, suggesting that both the polarity of the side-chain and the presence of a C-4 methyl substituent were crucial for trypanocidal activity.

**Figure 6:**
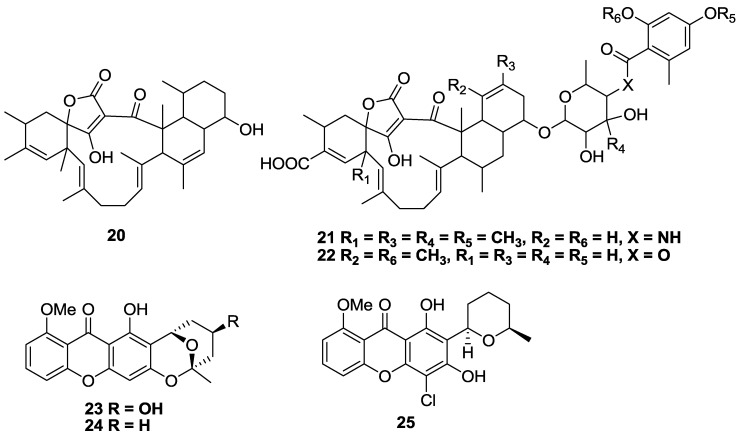
The structure of tetromycins (**20**–**22**) and xanthone analogues (**23**–**25**) of marine origin for which activity against *T.b. brucei* (**20**–**22**), *T.b. rhodesiense* (**23**–**24**), *T. cruzi* (**23**–**25**) has been described.

Tetronic acid-containing tetromycin B (**20**) as well as tetromycins 1 (**21**), and 3 (**22**) isolated from *Streptomyces axinellae* Po1001 cultivated from the Mediterranean sponge *Axinella polypoides*, showed limited activity against *T.b. brucei* with IC_50_ values of 34, 32, and 30 µM, respectively (Figure 6) [95]. Compounds **20** and **23** had poor selectivity (SI < 2) against 293T kidney cells, with the most selective compound **21,** having a SI > 3. Three new heterocyclic-substituted xanthone analogues (**23**–**25**) were isolated from the fungus *Chaetomium* sp. which was obtained from an algal species collected in Greece [96]. Of the series, compound **23** was the most active and selective for *T.b. rhodesiense* with an IC_50_ of 13.3 µM and a SI of 13 *versus* L6 cells. In contrast, the molecule had marginal activity against *T. cruzi* with an IC_50_ value >28 µM. Compound **25** had the greatest activity and selectivity against *T. cruzi* with an IC_50_ value of 3.8 µM and SI of 31, while **24** exhibited a similar activity against both parasites with IC_50_ values of 25 and 19 µM against *T. cruzi* and *T.b. brucei*, respectively, and a SI > 10 [96].

### 2.3. Alkaloids

**Figure 7:**
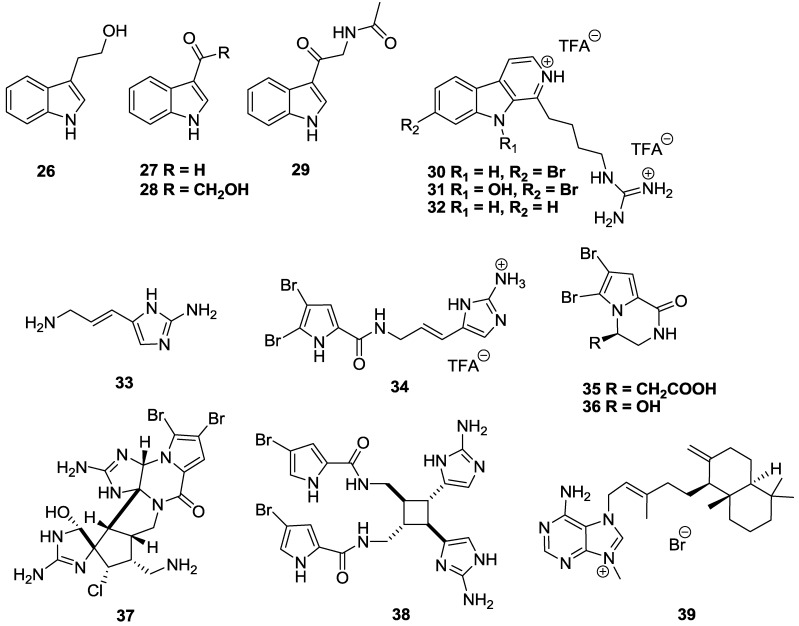
The structure of indole-, bromopyrrole-, and purine-based alkaloids of marine origin which have shown activity against *T.b. rhodesiense* (**26**, **30**–**38**), *T. cruzi* (**27**–**29**, **33**, **39**) and *T.b. brucei* (**39**).

A number of indole-, bromopyrrole-, and purine-based alkaloids have shown a range of anti-trypanosomal activity (Figure 7). An indole alkaloid tryptophol (**26**) isolated from the Turkish, Aegean Sea sponge *Ircinia spinulosa* [97] showed broad-spectrum inhibitory activity against a panel of parasitic protozoa, including *T.b. rhodesiense* with an IC_50_ value of 36.6 µM, while showing no significant toxicity against L6 cells (SI > 11) [88]. Three other indole alkaloids sourced from the marine bacterium *Bacillus pumilus*, isolated from a Panamanian collection of the black coral *Anthiphates* sp., namely 3-formylindole (**27**), 3-hydroxyacetylindole (**28**) and *N*-acetyl-β-oxotryptamine (**29**) showed marginal activity against *T. cruzi* (in a modification of the β-galactosidase method, whereby trypomastigotes are washed off before addition of compound to infected host cells) with IC_50_ values of 26.9, 20.6 and 19.4 µM, respectively, although the selectivity of the compounds was very poor (SI < 4) [98]. A New Zealand collection of the ascidian *Pseudodistoma opacum* yielded three alkylguanidine-substituted β-carboline alkaloids, opacalines A–C [99]. Opacaline A (**30**) and the *N*-hydroxy analogue opacaline B (**31**) showed marginal inhibition of *T.b. rhodesiense* with IC_50_ values of 30 and 27 µM, but had poor selectivity (<5). Compound **32**, a synthetically-prepared de-bromo analogue of **30** had improved activity against *T.b. rhodesiense* with an IC_50_ value of 12 µM and a slightly higher SI of 7 *versus* mammalian L6 cells [99].

The Turkish sponge, *Agelas oroides* collected in the Northern Aegean Sea yielded a series of bromopyrrole derivatives [89]. Moderate activity against both *T.b. rhodesiense* and *T. cruzi* with IC_50_ values of 17 and 18 µM, respectively, was observed for amino-1-(aminoimidazoyl)-prop-1-ene (**33**). However, the molecule displayed significant cytotoxicity towards L6 cells with an IC_50_ value of 5 µM. Oroidin trifluoroacetate salt (**34**) inhibited *T.b. rhodesiense* growth with an IC_50_ value of 25 µM, with no activity demonstrated against *T. cruzi* (IC_50_ > 62 µM) and L6 cells (IC_50_ = 157 µM). Bromopyrroles **35**–**38** sourced from another study of Turkish sponges belonging to the genera *Agelas* and *Axinella* displayed a range of activities against *T.b. rhodesiense* and *T. cruzi*, utilising an assay where *T.* *cruzi* trypomastigotes were washed off infected host cells before addition of compound [100]. The alkaloid longamide B (**35**) obtained from *Agelas dispar* [101] was active against *T.b. rhodesiense*, IC_50_ = 4.3 µM and displayed moderate cytotoxicity against L6 cells with an IC_50_ of 28 µM [100]. The compound displayed no activity against *T. cruzi* (IC_50_ > 94 µM). The hydroxyl analogue, longamide A (**36)** isolated from *Agelas longissima* [102] was over sixty-times less active against *T.b. rhodesiense* (IC_50_ > 290 µM) suggesting the importance of the carboxymethyl substituent for trypanocidal activity [100]. The oroidin dimer dibromopalau’amine, extracted from *Axinella verrucosa* [103], (**37**) exhibited sub-micromolar selective activity against *T.b. rhodesiense* with an IC_50_ value of 0.8 µM and a SI of 10 compared with mammalian L6 cells [100]. As with previous bromoryrroles, the compound had no activity against *T. cruzi* with an IC_50_ value of 119 µM. A second oroidin dimer, sceptrin (**38**), obtained from *Agelas sceptrum* [104] also showed selective activity against *T.b. rhodesiense* with an IC_50_ value of 15.7 µM and again no activity against *T. cruzi* (IC_50_ = 97 µM) or the mammalian L6 cell line (IC_50_ > 145 µM) [100]. Synthetically prepared agelasine D (**39**) a bicyclic diterpenoid purine, originally isolated from the Okinawan sea sponge *Agelas nakamurai* [105], inhibited both *T.b. brucei* and *T. cruzi* growth with IC_50_ values of 1.8 and 9 µM, respectively [106]. However, the selectivity of **39** was poor with an SI of <7 against MRC-5 human fetal lung fibroblasts cells.

**Figure 8:**
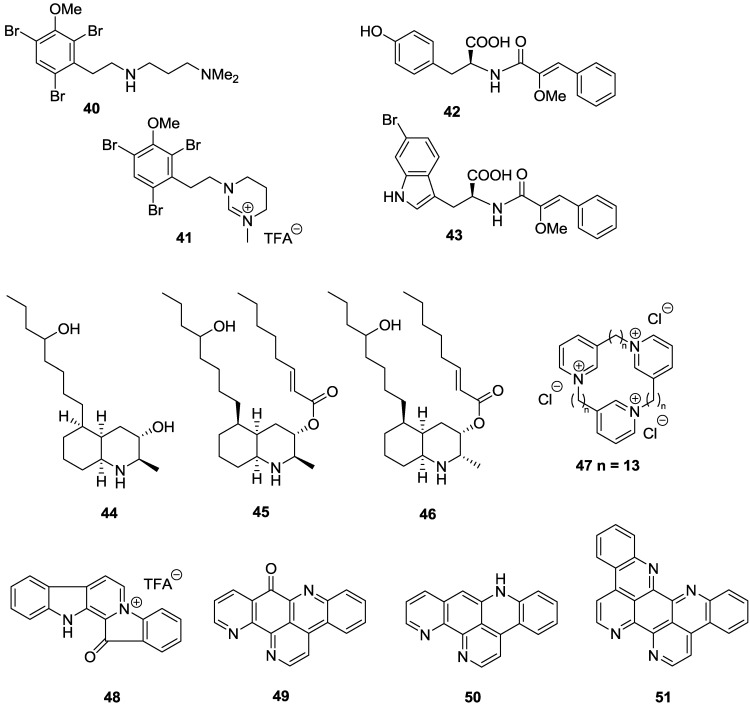
The structure of alkaloids of marine origin for which activity against *T.b. brucei* (**40**–**43**, **47**, **49**–**51**), *T.b. rhodesiense* (**44**–**46**, **48**) and *T. cruzi* (**45**, **46**) has been described.

Two brominated β-phenyl ethylamine-based alkaloids, convolutamines I (**40**) and J (**41**), were reported from a Tasmanian bryozoan *Amathia tortusa* with IC_50_ values against *T.b. brucei* of 1.1 and 13.7 µM, respectively (Figure 8) [107]. However, only convolutamine I (**40**) had a favorable SI of 18 against HEK-293 cells, with convolutamine J demonstrating cytotoxicity (SI > 3). As part of a HTS screen of a pre-fractionated natural product library to identify inhibitors of *T.b. brucei*, two cinnamoyl amino acids, iotrochotamides A (**42**) and B (**43**), were reported from an Australian marine sponge *Iotrochota* sp. [108]. Compounds **42** and **43** showed low micromolar activity against *T.b. brucei* with IC_50_ values of 3.4 and 4.7 µM, respectively, while exhibiting mild cytotoxicity against, HEK-293 with 85% inhibition at 50 µM for **42** and 100% inhibition at 70 µM for **43**. Decahydroquinoline alkaloids lepadins D–F (**44**–**46)**, were reported from a Great Barrier Reef collection of an ascidian *Didemnum* sp. [109]. Compounds **45** and **46** exhibited selective sub-micromolar activity against *T.b. rhodesiense* with IC_50_ values of 0.9 and 0.55 µM, respectively, and selectivity indices >40 *versus* mammalian L6 cells. Lepadins also displayed activity against *T. cruzi*, with IC_50_ values of 5.2 and 6.2 µM reported for **45** and **46**, but the SI was only 7 [109]. The presence of the 2E-octenoic acid ester functionality in **45** and **46** was concluded to be essential for the anti-trypanosomal activity of the series as the hydroxyl analogue **44** was observed to be over 20-fold less active against *T.b. rhodesiense* (IC_50_ = 19 µM) and was inactive against *T. cruzi* (IC_50_ = 125 µM). A synthetic preparation of a 3-alkylpyridinium alkaloid, viscosamine (**47**), originally isolated from the Arctic sponge *Haliclona viscosa* [110], displayed sub-micromolar, selective activity against *T.b. brucei* with an IC_50_ of 0.41 µM and SI of 63 against HEK-293 [111]. The pentacyclic bis-indole alkaloid fascaplysin (**48**) isolated from a Fijian collection of the sponge *Hyrtios* cf. *erecta* exhibited wide-ranging biological activity, including potent, selective activity against *T.b. rhodesiense* with an IC_50_ value of 0.46 μM and SI of 15 *versus* L6 cells [112]. Pyridoacridines ascididemnin (**49**) and 12-deoxyascididemnin (**50**), isolated from an Australian ascidian *Polysyncraton echinatum* also displayed selective sub-micromolar activity against *T.b. brucei* with IC_50_ values of 0.032 and 0.077 μM, respectively, and selectivity indices >45, against HEK-293 [113]. Eilatin (**51**) an analogue of ascididemnin was over 40 fold less active against *T.b. brucei* with an IC_50_ of 1.33 µM [113].

**Figure 9:**
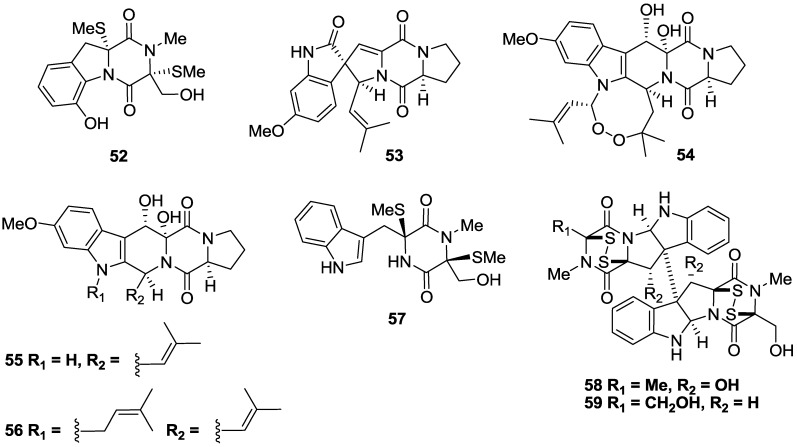
The structure of diketopiperazines of marine origin which have shown activity against *T.b. brucei* (**52**–**59**).

A series of dimethylthio (**52**), spiro-pentacyclic (**53**) and fused penta- and hexacyclic diketopiperazines (**54**–**56**) isolated from the marine-derived fungus *Aspergillus fumigatus* sourced from a Vanuatu sediment showed varying activity against *T.b. brucei* with IC_50_ values of 8.5, 5.7, 12.9, 6.4 and 19.5 µM, respectively [114] (Figure 9). The cytotoxicity of the compounds also varied with compounds **52** and **55** having a SI > 10, while **53**, **54** and **56** were considerably cytotoxic with SI < 8. A dimethylthio (**57**) and two disulfide diketopiperazines, verticilin B (**58**) and chaetocin (**59**) were isolated from the marine fungus *Nectria inventa* which was obtained from a dredge sample of deep-water Californian sediment [114]. Compound **57** had low micromolar, selective activity against *T.b. brucei* with an IC_50_ of 5.9 µM and SI of 16, while verticilin B (**58**) and chaetocin (**59**) exhibited potent, sub-micromolar activity against *T.b. brucei* with IC_50_ values of 0.007 and 0.002 μM, respectively [114]. However, the molecules exhibited pronounced cytotoxicity against Jurkat T Lymphocytes (IC_50_ < 0.6 µM) preventing further evaluation of their therapeutic potential.

**Figure 10:**
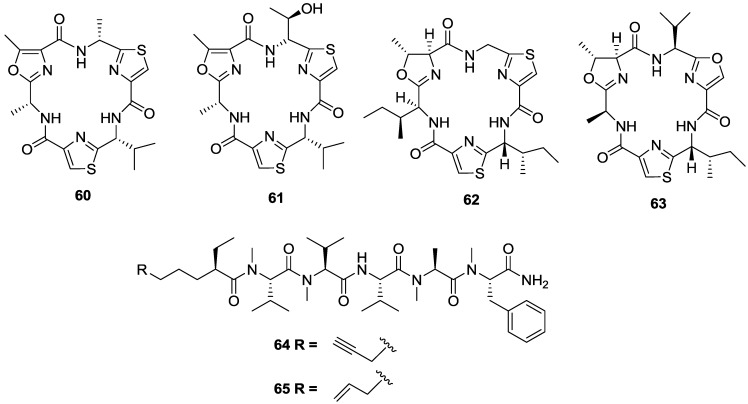
The structure of peptides of marine origin for which activity has been reported against *T. cruzi* (**60**–**61**), *T.b. rhodesiense* (**62**–**63**) and *T.b. brucei* (**64**–**65**).

Two cyclic hexapeptides, venturamides A (**60**) and B (**61**) were isolated from the Panamanian collection of the marine cyanobacterium *Oscillatoria* sp. [115] (Figure 10). The two compounds showed moderate activity against *T. cruzi* with IC_50_ values of 14.6 and 15.8 µM, respectively, and mild cytotoxicity to mammalian Vero (monkey kidney epithelial) cells with IC_50_ values of 86 and 56 µM, respectively, and thus an SI of < 6. Related cyclic peptides aerucyclamides B (**62**) and C (**63**) isolated from the cyanobacterium *Microrcystis aeruginosa* also displayed anti-trypanosomal activity with IC_50_ values of 15.9 and 9.2 µM, respectively, reported for *T.b. rhodesiense* [116]. Aerucyclamide C had a SI of 12 against L6 cells, whilst the SI of **62** was lower at 8. In a study using natural products as chemical probes to identify the molecular targets of small molecules, two linear peptides, almiramides B (**64**) and C (**65**) extracted from a Panamanian collection of the marine cyanobacterium *Lyngbya majuscula* were found to be low micromolar inhibitors of *T.b. brucei* with IC_50_ values of 6 and 3 µM, respectively [117]. Almiramide C displayed a SI of 11 compared to Vero cells while the SI for almiramide B was slightly lower at 9. Moreover, through a series of target based affinity probes, and fluorescence site localisation imaging studies, the compounds were shown to disrupt glycosome function in the parasite. Glycolysis is an essential pathway in trypanosomatids, and glycosomal enzymes have been identified as a potential drug target in trypanosomes [118].

## 3. Conclusions

A large number of structurally diverse marine natural products have been identified with trypanocidal activity. The manadoperoxides isolated from the marine sponge *Plakortis cfr. lita* are the most promising compounds for HAT. Manadoperoxide B (**12**) was the most active and selective molecule of the series exhibiting sub-micromolar activity against *T.b. rhodesiense* whilst highly selective against mammalian cells [94]. This compound was also demonstrated to possess anti-malarial activity, however, it is reported to be more than 700-fold less active against *Plasmodium falciparum* (D10) than *T.b. rhodesiense* [119]. As manadoperoxide B has sub-micromolar activity against *T.b. rhodesiense* and is not cytotoxic, one would anticipate that the physiochemical properties of the molecule, together with the biological activity are being investigated further to ensure the molecule possesses the required characteristics to meet the final target product profile.

The heterocyclic-substituted xanthone analogue **25** isolated from the marine fungus *Chaetomium* sp. was the most active and selective, marine derived compound for *T. cruzi* [96]. However, xanthones have been reported to have activity against multiple organisms and disease indications through interacting with a plethora of enzymes and targets [120]. This promiscuous activity may prevent further development of the compounds for Chagas disease.

In the last decade numerous molecules, both natural and synthetic, have been identified with trypanocidal activity. However, only two, have entered pre-clinical development for HAT. Furthermore, despite the identification of new targets and a multitude of *in vitro* and *in vivo* studies having been conducted, candidates for Chagas disease have failed to progress to the advanced stages of clinical development. Many of the molecules identified with potent trypanocidal activity, cannot be developed further as they possess unsuitable and undesirable structural and pharmacokinetic properties. This highlights the need to continue to explore other avenues for new chemical entities, whilst reviewing the approaches currently undertaken and the potential reasons for the lack of success. Evaluation of the current *in vitro* assays used to identify new compounds, in particular the life cycle stage for Chagas disease, is warranted. This is particularly true for the *in vivo* models where the parasite strain, administration route and duration of the study can impact on the outcomes.

Marine natural products have provided the pharmaceutical industry with many incredibly potent compounds—some developed into therapeutics whilst others providing valuable insights into the biology of disease and desired attributes of the compounds required to ameliorate it. Whilst compounds isolated from this source have yet to progress to pre-clinical development for trypanosomatid diseases, collectively the improvements to the *in vitro* assays used to identify them, the *in vivo* models used to evaluate them, and the methodology required for isolating them could change this situation.

## References

[1] Lozano R., Naghavi M., Foreman K., Lim S., Shibuya K., Aboyans V., Abraham J., Adair T., Aggarwal R., Ahn S.Y. (2012). Global and regional mortality from 235 causes of death for 20 age groups in 1990 and 2010: A systematic analysis for the global burden of disease study 2010. Lancet.

[2] Murray C.J.L., Vos T., Lozano R., Naghavi M., Flaxman A.D., Michaud C., Ezzati M., Shibuya K., Salomon J.A., Abdalla S. (2012). Disability-adjusted life years (DALYs) for 291 diseases and injuries in 21 regions, 1990–2010: A systematic analysis for the global burden of disease study 2010. Lancet.

[3] Pepin J., Meda H.A. (2001). The epidemiology and control of human African trypanosomiasis. Adv. Parasitol..

[4] Zeledon R., Rabinovich J.E. (1981). Chagas disease: An ecological appraisal with special emphasis on its insect vectors. Annu. Rev. Entomol..

[5] Simarro P.P., Diarra A., Ruiz Postigo J.A., Franco J.R., Jannin J.G. (2011). The human African trypanosomiasis control and surveillance programme of the World Health Organization 2000–2009: The way forward. PLoS Negl. Trop. Dis..

[6] Moncayo A., Silveira A.C. (2009). Current epidemiological trends for Chagas disease in Latin America and future challenges in epidemiology, surveillance and health policy. Mem. Inst. Oswaldo Cruz.

[7] Apted F.I.C., Mulligan H.W. (1970). Clinical manifestations and diagnosis of sleeping sickness. The African Trypanosomiases.

[8] Atouguia J.M., Kennedy P.G.E., Davis L.E., Davis L.E., Kennedy P.G.E. (2000). Neurological aspects of human African trypanosomiasis. Infectious Diseases of the Nervous System.

[9] Galfand M. (1947). Transitory neurological signs in sleeping sickness. Trans. R. Soc. Trop. Med. Hyg..

[10] Lundkvist G.B., Kristensson K., Bentivoglio M. (2004). Why trypanosomes cause sleeping sickness. Physiology.

[11] Human African trypanosomiasis (sleeping sickness). http://www.who.int/mediacentre/factsheets/fs259/en/.

[12] Rassi A., Rassi A., Marin-Neto J.A. (2010). Chagas disease. Lancet.

[13] Rassi A., Rezende J.M., Luquetti A.O., Telleria J., Tibayrenc M. (2010). Clinical phases and forms of Chagas disease. American Trypanosomiasis (Chagas Disease). One Hundred Years of Research.

[14] Munoz-Saravia S.G., Haberland A., Wallukat G., Schimke I. (2012). Chronic Chagas heart disease: A disease on its way to becoming a worldwide health problem: Epidemiology, etiopathology, treatment, pathogenesis and laboratory medicine. Heart Fail. Rev..

[15] Brun R., Blum J., Chappuis F., Burri C. (2010). Human African trypanosomiasis. Lancet.

[16] Pepin J., Milord F. (1994). The treatment of human African trypanosomiasis. Adv. Parasitol..

[17] Milord F., Pepin J., Loko L., Ethier L., Mpia B. (1992). Efficacy and toxicity of eflornithine for treatment of *Trypanosoma brucei gambiense* sleeping sickness. Lancet.

[18] Priotto G., Kasparian S., Ngouama D., Ghorashian S., Arnold U., Ghabri S., Karunakara U. (2007). Nifurtimox-eflornithine combination therapy for second-stage *Trypanosoma brucei gambiense* sleeping sickness: A randomized clinical trial in Congo. Clin. Infect. Dis..

[19] Priotto G., Kasparian S., Mutombo W., Ngouama D., Ghorashian S., Arnold U., Ghabri S., Baudin E., Buard V., Kazadi-Kyanza S. (2009). Nifurtimox-eflornithine combination therapy for second-stage African *Trypanosoma brucei gambiense* trypanosomiasis: A multicentre, randomised, phase III, non-inferiority trial. Lancet.

[20] Apt W. (2010). Current and developing therapeutic agents in the treatment of Chagas disease. Drug Des. Devel. Ther..

[21] Castro J.A., Diaz de Toranzo E.G. (1988). Toxic effects of nifurtimox and benznidazole, two drugs used against American trypanosomiasis (Chagas disease). Biomed. Environ. Sci..

[22] Jackson Y., Alirol E., Getaz L., Wolff H., Combescure C., Chappuis F. (2010). Tolerance and safety of nifurtimox in patients with chronic Chagas disease. Clin. Infect. Dis..

[23] Hasslocher-Moreno A.M., Do Brasil P.E., De Sousa A.S., Xavier S.S., Chambela M.C., Sperandio Da Silva G.M. (2012). Safety of benznidazole use in the treatment of chronic Chagas disease. J. Antimicrob. Chemother..

[24] Kaiser M., Bray M.A., Cal M., Bourdin Trunz B., Torreele E., Brun R. (2011). Antitrypanosomal activity of fexinidazole, a new oral nitroimidazole drug candidate for treatment of sleeping sickness. Antimicrob. Agents Chemother..

[25] Jacobs R.T., Nare B., Wring S.A., Orr M.D., Chen D., Sligar J.M., Jenks M.X., Noe R.A., Bowling T.S., Mercer L.T. (2011). SCYX-7158, an orally-active benzoxaborole for the treatment of stage 2 human African trypanosomiasis. PLoS Negl. Trop. Dis..

[26] Jacobs R.T., Nare B., Phillips M.A. (2011). State of the art in African trypanosome drug discovery. Curr. Top. Med. Chem..

[27] Barker R.H., Liu H., Hirth B., Celatka C.A., Fitzpatrick R., Xiang Y., Willert E.K., Phillips M.A., Kaiser M., Bacchi C.J. (2009). Novel S-adenosylmethionine decarboxylase inhibitors for the treatment of human African trypanosomiasis. Antimicrob. Agents Chemother..

[28] Bacchi C.J., Barker R.H., Rodriguez A., Hirth B., Rattendi D., Yarlett N., Hendrick C.L., Sybertz E. (2009). Trypanocidal activity of 8-methyl-5′-[(Z)-4-aminobut-2-enyl](methylamino)adenosine (Genz-644131), an adenosylmethionine decarboxylase inhibitor. Antimicrob. Agents Chemother..

[29] Price H.P., Menon M.R., Panethymitaki C., Goulding D., McKean P.G., Smith D.F. (2003). Myristoyl-CoA: Protein *N*-myristoyltransferase, an essential enzyme and potential drug target in kinetoplastid parasites. J. Biol. Chem..

[30] Frearson J.A., Brand S., McElroy S.P., Cleghorn L.A.T., Smid O., Stojanovski L., Price H.P., Guther M.L.S., Torrie L.S., Robinson D.A. (2010). *N*-myristoyltransferase inhibitors as new leads to treat sleeping sickness. Nature.

[31] Wyllie S., Oza S.L., Patterson S., Spinks D., Thompson S., Fairlamb A.H. (2009). Dissecting the essentiality of the bifunctional trypanothione synthetase-amidase in *Trypanosoma brucei* using chemical and genetic methods. Mol. Microbiol..

[32] Clayton J. (2010). Chagas disease: Pushing through the pipeline. Nature.

[33] A Study of the Use of Oral Posaconazole (POS) in the Treatment of Asymptomatic Chronic Chagas Disease. Clinical Trials.

[34] Chen C.K., Leung S.S., Guilbert C., Jacobson M.P., McKerrow J.H., Podust L.M. (2010). Structural characterization of CYP51 from *Trypanosoma cruzi* and *Trypanosoma brucei* bound to the antifungal drugs posaconazole and fluconazole. PLoS Negl. Trop. Dis..

[35] Gunatilleke S.S., Calvet C.M., Johnston J.B., Chen C.K., Erenburg G., Gut J., Engel J.C., Ang K.K., Mulvaney J., Chen S. (2012). Diverse inhibitor chemotypes targeting *Trypanosoma cruzi* CYP51. PLoS Negl. Trop. Dis..

[36] Lepesheva G.I., Villalta F., Waterman M.R. (2011). Targeting *Trypanosoma cruzi* sterol 14alpha-demethylase (CYP51). Adv. Parasitol..

[37] Soeiro Mde N., de Souza E.M., da Silva C.F., Batista Dda G., Batista M.M., Pavao B.P., Araujo J.S., Aiub C.A., da Silva P.B., Lionel J. (2013). *In vitro* and *in vivo* studies of the antiparasitic activity of sterol 14alpha-demethylase (CYP51) inhibitor VNI against drug-resistant strains of *Trypanosoma cruzi*. Antimicrob. Agents Chemother..

[38] Buckner F.S. (2008). Sterol 14-demethylase inhibitors for *Trypanosoma cruzi* infections. Adv. Exp. Med. Biol..

[39] Clayton J. (2010). The promise of *T. cruzi* genomics. Nature.

[40] Lisvane Silva P., Mantilla B.S., Barison M.J., Wrenger C., Silber A.M. (2011). The uniqueness of the *Trypanosoma cruzi* mitochondrion: Opportunities to identify new drug target for the treatment of Chagas disease. Curr. Pharm. Des..

[41] Soeiro M.N., de Castro S.L. (2009). *Trypanosoma cruzi* targets for new chemotherapeutic approaches. Expert Opin. Ther. Targets.

[42] Newman D.J., Cragg G.M. (2012). Natural products as sources of new drugs over the 30 years from 1981 to 2010. J. Nat. Prod..

[43] Blunt J.W., Copp B.R., Keyzers R.A., Munro M.H.G., Prinsep M.R. (2013). Marine natural products. Nat. Prod. Rep..

[44] Bergmann W., Feeney R.J. (1950). The isolation of a new thymine pentoside from sponges. J. Am. Chem. Soc..

[45] Swift A.N. (1951). Contributions to the study of marine products. Component acids of lipids of sponges. J. Org. Chem..

[46] O’Day D.M., Poirier R.H., Jones D.B., Elliott J.H. (1976). Vidarabine therapy of complicated *Herpes simplex* keratitis. Am. J. Ophthalmol..

[47] Pavan-Langston D., Hess F. (1977). Ocular and systemic antiviral activity of vidarabine. Compr. Ther..

[48] Mori J., Tsubokura M., Kami M. (2011). Cytarabine dose for acute myeloid leukemia. N. Engl. J. Med..

[49] Fox B.W. (1977). Pharmacology and chemistry of some inhibitors of herpes replication. J. Antimicrob. Chemother..

[50] Gedik C.M., Collins A.R. (1991). The mode of action of 1-beta-d-arabinofuranosylcytosine in inhibiting DNA repair; New evidence using a sensitive assay for repair DNA synthesis and ligation in permeable cells. Mutat. Res..

[51] Olivera B.M., Gray W.R., Zeikus R., McIntosh J.M., Varga J., Rivier J., De Santos V., Cruz L.J. (1985). Peptide neurotoxins from fish-hunting cone snails. Science.

[52] Miljanich G.P. (2004). Ziconotide: Neuronal calcium channel blocker for treating severe chronic pain. Curr. Med. Chem..

[53] Lovaza Drug Details. http://www.accessdata.fda.gov/scripts/cder/drugsatfda/index.cfm?fuseaction=Search.DrugDetails.

[54] Vascepa Food and Drug Administration Orange Book: Approved drug products with therapeutic equivalence evaluations. http://www.accessdata.fda.gov/scripts/cder/ob/docs/obdetail.cfm?Appl_No=202057&TABLE1=OB_Rx.

[55] Strobel C., Jahreis G., Kuhnt K. (2012). Survey of *n*-3 and *n*-6 polyunsaturated fatty acids in fish and fish products. Lipids Health Dis..

[56] Nestel P.J., Connor W.E., Reardon M.F., Connor S., Wong S., Boston R. (1984). Suppression by diets rich in fish oil of very low-density lipoprotein production in man. J. Clin. Invest..

[57] Sanders T.A.B., Sullivan D.R., Reeve J., Thompson G.R. (1985). Triglyceride-lowering effect of marine polyunsaturates in patients with hypertriglyceridemia. Arteriosclerosis.

[58] Bordin P., Bodamer O.A.F., Venkatesan S., Gray R.M., Bannister P.A., Halliday D. (1998). Effects of fish oil supplementation on apolipoprotein B100 production and lipoprotein metabolism in normolipidaemic males. Eur. J. Clin. Nutr..

[59] Madsen L., Rustan A.C., Vaagenes H., Berge K., Dyroy E., Berge R.K. (1999). Eicosapentaenoic and docosahexaenoic acid affect mitochondrial and peroxisomal fatty acid oxidation in relation to substrate preference. Lipids.

[60] Davidson M.H. (2006). Mechanisms for the hypotriglyceridemic effect of marine omega-3 fatty acids. Am. J. Cardiol..

[61] Hirata Y., Uemura D. (1986). Halichondrins—Antitumor polyether macrolides from a marine sponge. Pure Appl. Chem..

[62] Kuznetsov G., Towle M.J., Cheng H.S., Kawamura T., TenDyke K., Liu D., Kishi Y., Yu M.J., Littlefield B.A. (2004). Induction of morphological and biochemical apoptosis following prolonged mitotic blockage by halichondrin B macrocyclic ketone analog E7389. Cancer Res..

[63] Jordan M.A., Kamath K., Manna T., Okouneva T., Miller H.P., Davis C., Littlefield B.A., Wilson L. (2005). The primary antimitotic mechanism of action of the synthetic halichondrin E7389 is suppression of microtubule growth. Mol. Cancer Ther..

[64] Dabydeen D.A., Burnett J.C., Bai R.L., Verdier-Pinard P., Hickford S.J.H., Pettit G.R., Blunt J.W., Munro M.H.G., Gussio R., Hamel E. (2006). Comparison of the activities of the truncated halichondrin B analog NSC 707389 (E7389) with those of the parent compound and a proposed binding site on tubulin. Mol. Pharmacol..

[65] Francisco J.A., Cerveny C.G., Meyer D.L., Mixan B.J., Klussman K., Chace D.F., Rejniak S.X., Gordon K.A., DeBlanc R., Toki B.E. (2003). cAC10-vcMMAE, an anti-CD30–monomethyl auristatin E conjugate with potent and selective antitumor activity. Blood.

[66] Pettit G.R., Kamano Y., Herald C.L., Tuinman A.A., Boettner F.E., Kizu H., Schmidt J.M., Baczynskyj L., Tomer K.B., Bontems R.J. (1987). The isolation and structure of a remarkable marine animal antineoplastic constituent: Dolastatin 10. J. Am. Chem. Soc..

[67] Deng C., Pan B., O’Connor O.A. (2013). Brentuximab vedotin. Clin. Cancer Res..

[68] Rinehart K.L., Holt T.G., Fregeau N.L., Stroh J.G., Keifer P.A., Sun F., Li L.H., Martin D.G. (1990). Ecteinascidins 729, 743, 745, 759A, 759B, and 770: Potent antitumor agents from the Caribbean tunicate *Ecteinascidia turbinata*. J. Org. Chem..

[69] Zewail-Foote M., Hurley L.H. (2001). Differential rates of reversibility of ecteinascidin 743-DNA covalent adducts from different sequences lead to migration to favored bonding sites. J. Am. Chem. Soc..

[70] Takebayashi Y., Pourquier P., Zimonjic D.B., Nakayama K., Emmert S., Ueda T., Urasaki Y., Kanzaki A., Akiyama S., Popescu N. (2001). Antiproliferative activity of ecteinascidin 743 is dependent upon transcription-coupled nucleotide-excision repair. Nat. Med..

[71] Soares D.G., Escargueil A.E., Poindessous V., Sarasin A., De Gramont A., Bonatto D., Henriques J.A.P., Larsen A.K. (2007). Replication and homologous recombination repair regulate DNA double-strand break formation by the antitumor alkylator ecteinascidin 743. Proc. Natl. Acad. Sci. USA..

[72] Herrero A.B., Martin-Castellanos C., Marco E., Gago F., Moreno S. (2006). Cross-talk between nucleotide excision and homologous recombination DNA repair pathways in the mechanism of action of antitumor trabectedin. Cancer Res..

[73] Gerwick W.H., Moore B.S. (2012). Lessons from the past and charting the future of marine natural products drug discovery and chemical biology. Chem. Biol..

[74] Jones A.J., Avery V.M. (2013). Whole-organism high-throughput screening against *Trypanosoma brucei brucei*. Exp. Opin. Drug Discov..

[75] Sykes M.L., Avery V.M. (2013). Approaches to protozoan drug discovery: Phenotypic screening. J. Med. Chem..

[76] Stevens J., Brisse S., Maudlin I., Holmes P.H., Miles M.A. (2004). Systematics of trypanosomes of medical and veterinary importance. The Trypanosomiases.

[77] Pink R., Hudson A., Mouries M.A., Bendig M. (2005). Opportunities and challenges in antiparasitic drug discovery. Nat. Rev. Drug Discov..

[78] Chennamaneni N.K., Arif J., Buckner F.S., Gelb M.H. (2009). Isoquinoline-based analogs of the cancer drug clinical candidate tipifarnib as anti-*Trypanosoma cruzi* agents. Bioorg. Med. Chem. Lett..

[79] Romanha A.J., Castro S.L., Soeiro Mde N., Lannes-Vieira J., Ribeiro I., Talvani A., Bourdin B., Blum B., Olivieri B., Zani C. (2010). *In vitro* and *in vivo* experimental models for drug screening and development for Chagas disease. Mem. Inst. Oswaldo Cruz.

[80] Ennes-Vidal V., Menna-Barreto R.F., Santos A.L., Branquinha M.H., d’Avila-Levy C.M. (2010). Effects of the calpain inhibitor MDL28170 on the clinically relevant forms of *Trypanosoma cruzi in vitro*. J. Antimicrob. Chemother..

[81] Buckner F.S., Verlinde C.L., La Flamme A.C., Van Voorhis W.C. (1996). Efficient technique for screening drugs for activity against *Trypanosoma cruzi* using parasites expressing beta-galactosidase. Antimicrob. Agents Chemother..

[82] Vickerman K. (1985). Developmental cycles and biology of pathogenic trypanosomes. Br. Med. Bull..

[83] Da Silva A.J., Moser M. Trypanosomiasis, American (Chagas disease, *Trypanosoma cruzi*). Center for Disease Control and Prevention: Public Health Image Library (PHIL).

[84] Nwaka S., Hudson A. (2006). Innovative lead discovery strategies for tropical diseases. Nat. Rev. Drug Discov..

[85] Dardonville C., Fernandez-Fernandez C., Gibbons S.L., Jagerovic N., Nieto L., Ryan G., Kaiser M., Brun R. (2009). Antiprotozoal activity of 1-phenethyl-4-aminopiperidine derivatives. Antimicrob. Agents Chemother..

[86] Jones D.C., Hallyburton I., Stojanovski L., Read K.D., Frearson J.A., Fairlamb A.H. (2010). Identification of a κ-opioid agonist as a potent and selective lead for drug development against human African trypanosomiasis. Biochem. Pharmacol..

[87] Sykes M.L., Baell J.B., Kaiser M., Chatelain E., Moawad S.R., Ganame D., Ioset J.R., Avery V.M. (2012). Identification of compounds with anti-proliferative activity against *Trypanosoma brucei brucei* strain 427 by a whole cell viability based HTS campaign. PLoS Negl. Trop. Dis..

[88] Orhan I., Sener B., Kaiser M., Brun R., Tasdemir D. (2010). Inhibitory activity of marine sponge-derived natural products against parasitic protozoa. Mar. Drugs.

[89] Tasdemir D., Topaloglu B., Perozzo R., Brun R., O’Neill R., Carballeira N.M., Zhang X., Tonge P.J., Linden A., Ruedi P. (2007). Marine natural products from the Turkish sponge *Agelas oroides* that inhibit the enoyl reductases from *Plasmodium falciparum*, *Mycobacterium tuberculosis* and *Escherichia coli*. Bioorg. Med. Chem..

[90] Regalado E.L., Tasdemir D., Kaiser M., Cachet N., Amade P., Thomas O.P. (2010). Antiprotozoal steroidal saponins from the marine sponge *Pandaros acanthifolium*. J. Nat. Prod..

[91] Regalado E.L., Jimenez-Romero C., Genta-Jouve G., Tasdemir D., Amade P., Nogueiras C., Thomas O.P. (2011). Acanthifoliosides, minor steroidal saponins from the Caribbean sponge *Pandaros acanthifolium*. Tetrahedron.

[92] Kossuga M.H., Nascimento A.M., Reimao J.Q., Tempone A.G., Taniwaki N.N., Veloso K., Ferreira A.G., Cavalcanti B.C., Pessoa C., Moraes M.O. (2008). Antiparasitic, antineuroinflammatory, and cytotoxic polyketides from the marine sponge *Plakortis angulospiculatus* collected in Brazil. J. Nat. Prod..

[93] Feng Y.J., Davis R.A., Sykes M., Avery V.M., Camp D., Quinn R.J. (2010). Antitrypanosomal cyclic polyketide peroxides from the Australian marine sponge *Plakortis* sp.. J. Nat. Prod..

[94] Chianese G., Fattorusso E., Scala F., Teta R., Calcinai B., Bavestrello G., Dien H.A., Kaiser M., Tasdemir D., Taglialatela-Scafati O. (2012). Manadoperoxides, a new class of potent antitrypanosomal agents of marine origin. Org. Biomol. Chem..

[95] Pimentel-Elardo S.M., Buback V., Gulder T.A.M., Bugni T.S., Reppart J., Bringmann G., Ireland C.M., Schirmeister T., Hentschel U. (2011). New tetromycin derivatives with anti-trypanosomal and protease inhibitory activities. Mar. Drugs.

[96] Pontius A., Krick A., Kehraus S., Brun R., Konig G.M. (2008). Antiprotozoal activities of heterocyclic-substituted xanthones from the marine-derived fungus *Chaetomium* sp.. J. Nat. Prod..

[97] Erdogan I., Sener B., Higa T. (2000). Tryptophol, a plant auxin isolated from the marine sponge *Ircinia spinulosa*. Biochem. Syst. Ecol..

[98] Martinez-Luis S., Gomez J.F., Spadafora C., Guzman H.M., Gutierrez M. (2012). Antitrypanosomal alkaloids from the marine bacterium *Bacillus pumilus*. Molecules.

[99] Chan S.T.S., Pearce A.N., Page M.J., Kaiser M., Copp B.R. (2011). Antimalarial β-carbolines from the New Zealand ascidian *Pseudodistoma opacum*. J. Nat. Prod..

[100] Scala F., Fattorusso E., Menna M., Taglialatela-Scafati O., Tierney M., Kaiser M., Tasdemir D. (2010). Bromopyrrole alkaloids as lead compounds against protozoan parasites. Mar. Drugs.

[101] Cafieri F., Fattorusso E., Taglialatela-Scafati O. (1998). Novel bromopyrrole alkaloids from the sponge *Agelas dispar*. J. Nat. Prod..

[102] Cafieri F., Fattorusso E., Mangoni A., Taglialatelascafati O. (1995). Longamide and 3,7-dimethylisoguanine, 2 novel alkaloids from the marine sponge *Agelas longissima*. Tetrahedron Lett..

[103] Aiello A., D’Esposito M., Fattorusso E., Menna M., Muller W.E.G., Perovic-Ottstadt S., Schroder H.C. (2006). Novel bioactive bromopyrrole alkaloids from the Mediterranean sponge *Axinella verrucosa*. Bioorg. Med. Chem..

[104] Walker R.P., Faulkner D.J., Van Engen D., Clardy J. (1981). Sceptrin, an antimicrobial agent from the sponge *Agelas sceptrum*. J. Am. Chem. Soc..

[105] Wu H., Nakamura H., Kobayashi J., Kobayashi M., Ohizumi Y., Hirata Y. (1986). Structures of agelasines, diterpenes having a 9-methyladeninium chromophore isolated from the Okinawan marine sponge *Agelas nakamurai hoshino*. Bull. Chem. Soc. Jpn..

[106] Vik A., Proszenyak A., Vermeersch M., Cos P., Maes L., Gundersen L.L. (2009). Screening of agelasine D and analogs for inhibitory activity against pathogenic protozoa; Identification of hits for visceral leishmaniasis and Chagas disease. Molecules.

[107] Davis R.A., Sykes M., Avery V.M., Camp D., Quinn R.J. (2011). Convolutamines I and J, antitrypanosomal alkaloids from the bryozoan *Amathia tortusa*. Bioorg. Med. Chem..

[108] Feng Y.J., Davis R.A., Sykes M.L., Avery V.M., Quinn R.J. (2012). Iotrochamides A and B, antitrypanosomal compounds from the Australian marine sponge *Iotrochota* sp.. Bioorg. Med. Chem. Lett..

[109] Wright A.D., Goclik E., Koenig G.M., Kaminsky R. (2002). Lepadins D–F: Antiplasmodial and antitrypanosomal decahydroquinoline derivatives from the tropical marine tunicate *Didemnum* sp.. J. Med. Chem..

[110] Volk C.A., Kock M. (2003). Viscosamine: The first naturally occurring trimeric 3-alkyl pyridinium alkaloid. Org. Lett..

[111] Rodenko B., Al-Salabi M.I., Teka I.A., Ho W., El-Sabbagh N., Ali J.A.M., Ibrahim H.M.S., Wanner M.J., Koomen G., De Koning H.P. (2011). Synthesis of marine-derived 3-alkylpyridinium alkaloids with potent antiprotozoal activity. ACS Med. Chem. Lett..

[112] Kirsch G., Konig G.M., Wright A.D., Kaminsky R. (2000). A new bioactive sesterterpene and antiplasmodial alkaloids from the marine sponge *Hyrtios* cf. *erecta*. J. Nat. Prod..

[113] Feng Y.J., Davis R.A., Sykes M.L., Avery V.M., Carroll A.R., Camp D., Quinn R.J. (2010). Antitrypanosomal pyridoacridine alkaloids from the Australian ascidian *Polysyncraton echinatum*. Tetrahedron Lett..

[114] Watts K.R., Ratnam J., Ang K.H., Tenney K., Compton J.E., McKerrow J., Crews P. (2010). Assessing the trypanocidal potential of natural and semi-synthetic diketopiperazines from two deep water marine-derived fungi. Bioorg. Med. Chem..

[115] Linington R.G., Gonzalez J., Urena L.D., Romero L.I., Ortega-Barría E., Gerwick W.H. (2007). Venturamides A and B: Antimalarial constituents of the Panamanian marine cyanobacterium *Oscillatoria* sp.. J. Nat. Prod..

[116] Portmann C., Blom J.F., Kaiser M., Brun R., Juttner F., Gademann K. (2008). Isolation of aerucyclamides C and D and structure revision of microcyclamide 7806A: Heterocyclic ribosomal peptides from *Microcystis aeruginosa* PCC 7806 and their antiparasite evaluation. J. Nat. Prod..

[117] Sanchez L.M., Knudsen G.M., Helbig C., De Muylder G., Mascuch S.M., Mackey Z.B., Gerwick L., Clayton C., McKerrow J.H., Linington R.G. (2013). Examination of the mode of action of the almiramide family of natural products against the kinetoplastid parasite *Trypanosoma brucei*. J. Nat. Prod..

[118] Verlinde C.L., Hannaert V., Blonski C., Willson M., Perie J.J., Fothergill-Gilmore L.A., Opperdoes F.R., Gelb M.H., Hol W.G., Michels P.A. (2001). Glycolysis as a target for the design of new anti-trypanosome drugs. Drug Resist. Updat..

[119] Fattorusso C., Persico M., Calcinai B., Cerrano C., Parapini S., Taramelli D., Novellino E., Romano A., Scala F., Fattorusso E. (2010). Manadoperoxides A–D from the Indonesian sponge *Plakortis* cfr. *simplex*. Further insights on the structure-activity relationships of simple 1,2-dioxane antimalarials. J. Nat. Prod..

[120] El-Seedi H.R., El-Barbary M.A., El-Ghorab D.M.H., Bohlin L., Borg-Karlson A.K., Goransson U., Verpoorte R. (2010). Recent insights into the biosynthesis and biological activities of natural xanthones. Curr. Med. Chem..

